# The influence of family socioeconomic status on physics achievement among Chinese high school students: the mediating roles of cultural capital and educational expectations

**DOI:** 10.3389/fpsyg.2026.1897951

**Published:** 2026-10-21

**Authors:** Liwei Chen, Jiajun Wu

**Affiliations:** 1 School of Mechanical and Electrical Engineering, Sanming University, Sanming, China; 2 School of Economics and Management, Sanming University, Sanming, China

**Keywords:** Chinese high school students, cultural capital, family socioeconomic status, parental educational expectations, physics achievement, stem education

## Abstract

Equitable participation in STEM education remains difficult when students have unequal access to family educational resources, and these disparities may be especially consequential in physics because the subject requires sustained conceptual and mathematical engagement. Drawing on Bourdieu's cultural capital theory and Eccles' expectancy-value theory, this study examined whether cultural capital and parental educational expectations mediate the relationship between family SES and physics achievement among Chinese high school students. A convenience sample of 226 Grade 10–12 students was recruited from one public high school in Fujian Province, China. Confirmatory factor analysis, hierarchical regression, bootstrap mediation, and moderation analyses were conducted in Stata 17. The results showed that family SES positively associated with physics achievement. Cultural capital and parental educational expectations statistically mediated this relationship. The positive SES-achievement association was stronger among rural students than among urban students. By integrating a material-resource pathway with a motivational pathway in a subject-specific model, this study extends research on family background to physics education in a Chinese high school context.

## 1 Introduction

Physics education plays a foundational role in the STEM (Science, Technology, Engineering, and Mathematics) talent pipeline because it develops students' scientific literacy, quantitative reasoning, and analytical problem-solving abilities (Maltese and Tai, 2011). It is also a demanding gateway subject: a recent study covering 555 U.S. high schools and 475,539 students linked school-level poverty with participation and performance in the physical sciences, underscoring the close relationship between socioeconomic conditions and access to STEM pathways (Hatzfeld et al., 2025). In China, physics is not only a compulsory subject for science-track students but also a key determinant of educational and career opportunities within the highly competitive National College Entrance Examination (Gaokao) system (Wang and Degol, 2013). Despite its importance, physics is widely perceived as one of the most difficult school subjects because it requires abstract reasoning, mathematical fluency, and conceptual understanding (Angell et al., 2004). Understanding the family and motivational factors that influence students' physics achievement is therefore both theoretically important and practically significant.

A substantial body of research has consistently identified family socioeconomic status (SES) as one of the strongest predictors of academic achievement across educational stages and subject domains (Sirin, 2005; White, 1982). Family SES is commonly measured through parental education, occupational status, and household income, which together reflect the educational, economic, and social resources available within the family (Bradley and Corwyn, 2002). Recent Chinese evidence continues to show that socioeconomic inequalities are associated with achievement. For example, a quasi-experimental study found that the transition to online education widened the performance gap between high- and low-SES students by 16.7%, with high-SES students gaining 0.464 grade-point units relative to disadvantaged students (Sun et al., 2024). These findings are consistent with the family investment model, which emphasizes unequal access to learning materials, extracurricular activities, private tutoring, and cognitively stimulating environments that facilitate academic success (Conger and Donnellan, 2007). In contrast, lower-SES families may face economic constraints that limit educational opportunities and increase family stress, thereby negatively influencing children's learning and development (Conger et al., 2002).

Although the relationship between family SES and general academic achievement has been extensively examined, considerably less research has focused on domain-specific achievement, particularly physics achievement. Physics differs from many other school subjects because it requires both conceptual abstraction and mathematical application. Students' success in physics may therefore depend heavily on access to educational resources, science-related learning opportunities, and family academic support. Consequently, the mechanisms linking family SES to physics achievement may differ from those underlying general academic performance.

Bourdieu's (1986) theory of cultural capital provides an important sociological perspective for understanding how family background shapes educational inequality. Cultural capital refers to the knowledge, skills, dispositions, and educational resources transmitted through family environments that align with the values and expectations of educational institutions (Lareau, 2011). In science education contexts, cultural capital may include science-related books, participation in science activities, familiarity with scientific discourse, and parental encouragement toward science learning (Archer et al., 2012). Previous studies have shown that science-related cultural capital positively predicts science aspirations, science identity, self-efficacy, and academic achievement (Aschbacher et al., 2010; DeWitt et al., 2016). Students from families with richer science-related cultural resources are more likely to perceive science as accessible, meaningful, and personally relevant, whereas students from disadvantaged backgrounds may experience weaker alignment between home environments and school science culture (Claussen and Osborne, 2013). Recent evidence from 1,036 Chinese high school students found that institutionalized and objectified cultural capital, including parental education and household book collections, was positively associated with school achievement (Jin et al., 2024). Such findings support closer examination of science-related cultural capital in physics learning.

In addition to cultural resources, parental educational expectations may also play a critical role in explaining how family SES influences students' physics achievement. According to expectancy-value theory, parents' beliefs and expectations are related to children's motivation, self-concept, task value, and academic persistence (Eccles and Wigfield, 2002). Parents with high educational expectations are more likely to monitor academic progress, encourage effort, communicate the importance of achievement, and provide emotional and educational support (Shen et al., 2025; Simpkins et al., 2012; Song et al., 2024; Ye et al., 2024). Empirical evidence demonstrates that parental educational expectations positively predict adolescents' academic motivation, engagement, and achievement across multiple educational domains (Davis-Kean, 2005; Jodl et al., 2001). In physics learning, where students frequently encounter difficulty and frustration, parental expectations may be particularly relevant to persistence and confidence.

The Chinese educational context further highlights the importance of examining these relationships. China's examination-oriented educational system places strong emphasis on academic performance as a pathway to social mobility (Fong, 2004). At the same time, substantial urban-rural disparities persist in educational resources, school quality, and extracurricular learning opportunities (Hannum and Park, 2007). Urban families generally possess greater access to tutoring institutions, science museums, libraries, and enrichment programs, whereas rural families often face structural disadvantages in educational support and science learning opportunities (Li et al., 2017). An analysis of 16 million Chinese college applicants found that an English-listening testing requirement increased the rural-urban gap in college access by 30% (Li et al., 2024). More recent longitudinal evidence from Chinese high-school students indicates that family SES accounts for a substantial part of rural disadvantage, while family cultural activities and parental educational expectations contribute differently to the observed gap (Hu, 2025). These findings suggest that the strength of the association between family SES and physics achievement may vary across residential contexts.

Despite growing interest in educational inequality and STEM achievement, several important gaps remain in the literature. First, existing research primarily focuses on general academic performance rather than subject-specific achievement, especially physics achievement. Second, limited research has examined science-related cultural capital as a mediator of the SES and physics achievement relationship. Third, few studies have integrated cultural capital theory and expectancy-value theory to examine material-resource and motivational pathways simultaneously in a non-Western physics-education context.

To address these gaps, the present study investigates how family SES influences physics achievement among Chinese high school students. It proposes an integrated framework in which cultural capital and parental educational expectations statistically mediate the relationship between family SES and physics achievement. In addition, this study explores urban-rural differences in the SES-achievement relationship.

## 2 Theoretical analysis and hypothesis development

### 2.1 Family socioeconomic status and physics achievement

Family socioeconomic status reflects the educational, occupational, and economic resources available within families. According to the family investment model, higher-SES families possess greater capacity to provide educational support, learning materials, extracurricular opportunities, and cognitively stimulating home environments that facilitate children's academic development (Conger and Donnellan, 2007). These advantages may be especially important in physics education, where students often require additional conceptual explanation, mathematical support, and extended practice opportunities.

Previous studies consistently demonstrate that students from higher-SES families achieve better academic outcomes than students from disadvantaged backgrounds (Sirin, 2005; White, 1982). In the Chinese educational context, SES-related educational inequalities remain substantial because family resources strongly influence access to educational opportunities and academic support (Zhang et al., 2015). Physics learning may be particularly sensitive to SES differences because the subject requires abstract reasoning, sustained effort, and high levels of cognitive engagement.

Students from higher-SES families are more likely to have access to science learning resources, supplementary tutoring, and supportive educational environments that directly contribute to physics achievement. By contrast, lower-SES students may encounter greater barriers in accessing educational support and developing effective learning strategies. Recent studies likewise identify SES-related disparities in Chinese adolescents' achievement and in access to educational resources (Xu and Jin, 2024; Shi et al., 2026; Sun et al., 2024). Therefore, the present study proposes the following hypothesis:

H1: Family socioeconomic status is positively associated with physics achievement among Chinese high school students.

### 2.2 The mediating role of cultural capital

Bourdieu's (1986) cultural capital theory argues that educational inequality is reproduced through unequal access to culturally valued resources and practices. Cultural capital includes educational resources, learning habits, cultural knowledge, and dispositions that align with school expectations and facilitate academic success (Lareau, 2011). In physics education, domain-specific cultural capital includes tutoring resources, time devoted to physics study, participation in science exhibitions, and access to science books. These resources are closely connected to the demands of physics learning because they provide repeated exposure to scientific concepts, structured practice, and opportunities to connect abstract content with observable phenomena (Archer et al., 2012). Their unequal distribution across SES groups therefore makes cultural capital a theoretically relevant mediator of the SES-physics-achievement relationship (Jin et al., 2024).

Such cultural resources may influence physics achievement through several mechanisms. Science books and tutoring provide additional exposure to scientific concepts; sustained study time supports practice and self-regulation; and science exhibitions connect formal learning with informal scientific experiences. Together, these indicators represent a material-resource pathway through which family SES may be linked with physics achievement.

Previous research has demonstrated that science capital significantly predicts science aspirations, science identity, and academic performance (DeWitt et al., 2016). However, relatively few studies have specifically examined whether cultural capital mediates the relationship between family SES and physics achievement. Accordingly, this study proposes the following hypothesis:

H2: Cultural capital mediates the relationship between family socioeconomic status and physics achievement.

### 2.3 The mediating role of parental educational expectations

Expectancy-value theory emphasizes that parents' beliefs and expectations are related to children's motivation, self-concept, effort, and academic achievement (Eccles and Wigfield, 2002). This pathway is especially relevant to physics because students must persist through abstract concepts, mathematical demands, and repeated problem solving. Parents with stronger educational expectations may communicate the importance of academic success, monitor learning progress, and provide emotional or instructional support (Simpkins et al., 2012). Recent Chinese evidence links parental educational expectations with engagement and academic success, while also showing that their meaning depends on family and student contexts (Song et al., 2024; Shen et al., 2025).

In the Chinese educational context, educational achievement is widely regarded as a major pathway to upward social mobility, which further strengthens the influence of parental educational expectations (Fong, 2004). Parents from higher-SES families often possess stronger educational aspirations and greater capacity to express them through monitoring, encouragement, and educational support. Longitudinal evidence on Chinese left-behind children also identifies parental educational expectations and supportive educational environments as predictors of subsequent academic success (Ye et al., 2024).

In physics learning, parental educational expectations may be especially important because students frequently experience conceptual difficulty, frustration, and academic pressure. High parental expectations may encourage students to persist when facing challenging physics tasks and devote greater effort to physics study. Previous studies consistently report positive relationships between parental educational expectations and students' academic achievement (Davis-Kean, 2005; Jodl et al., 2001). Nevertheless, relatively limited evidence exists regarding the mediating role of parental expectations in the relationship between family SES and physics achievement.

Cultural capital and parental educational expectations were selected together because they represent complementary dimensions of family advantage. Cultural capital captures the material-resource pathway emphasized by Bourdieu, whereas parental expectations capture the motivational pathway emphasized by expectancy-value theory. Examining both mediators in parallel makes it possible to assess whether SES is linked with physics achievement through access to domain-specific resources, through family motivational support, or through both pathways.

Therefore, this study proposes the following hypothesis:

H3: Parental educational expectations mediate the relationship between family socioeconomic status and physics achievement.

### 2.4 Urban-rural differences in the SES-achievement relationship

Educational inequality between urban and rural regions remains a major issue in China's educational system (Hannum and Park, 2007). Students in urban generally benefit from stronger educational infrastructure, more qualified teachers, richer extracurricular opportunities, and greater access to science-related learning resources. Rural students, by contrast, often face limited educational support, fewer science learning opportunities, and shortages of high-quality educational resources (Li et al., 2017).

Under conditions of resource scarcity, family socioeconomic resources may become particularly important in shaping students' educational experiences. Rural students from higher-SES families may receive substantially greater educational advantages compared with their lower-SES peers because family resources can compensate for deficiencies in school and community support. In contrast, urban students may obtain educational support from broader institutional and community resources even when family SES is relatively limited.

As a result, the association between family SES and physics achievement may be stronger among rural students than among urban students. Therefore, the present study proposes the following hypothesis:

H4: Urban vs. rural residence moderates the relationship between family socioeconomic status and physics achievement, such that the relationship is stronger among rural students.

## 3 Materials and methods

### 3.1 Participants

A convenience sampling design was used to recruit 226 Grade 10–12 students from one public high school in Fujian Province, China. Eligible participants were enrolled at the school, were present during data collection, and provided assent together with informed consent from a legal guardian. Female students comprised 48.2% of the sample, and the average age was 16.2 years (SD = 0.8). Students were enrolled in Grade 10 (34.5%), Grade 11 (36.3%), or Grade 12 (29.2%). Residential backgrounds included rural areas (39.4%), and urban districts (60.6%). For the moderation analysis, rural residence was coded 1, while urban residence was coded 0. Participation was voluntary and anonymous. The single-school convenience sample should not be interpreted as representative of all Chinese senior high school students.

### 3.2 Measures

All measures were administered as part of a self-report questionnaire specifically designed for this study. Items were developed based on established instruments in the family-achievement literature and adapted for the Chinese high school and physics education context.

#### 3.2.1 Family socioeconomic status

Family SES was assessed using three indicators: parental education, parental occupation, and household monthly income. Parental education was measured separately for fathers and mothers on a 5-point scale (1 = junior high school or below, 2 = senior high school, 3 = associate degree, 4 = bachelor degree, 5 = master degree or above). Parental occupation was recorded descriptively using the Chinese standard occupational classification but was excluded from the final SES composite because it did not load adequately with parental education and household income in the measurement model. Previous research recognizes occupation as a dimension of SES, although its exclusion from the present composite was based on our measurement results (Bradley and Corwyn, 2002; White, 1982). Household monthly income was reported on a 5-point scale: (1) below 5,000 CNY, (2) 5,000 to 8,000 CNY, (3) 8,000 to 15,000 CNY, (4) 15,000 to 30,000 CNY, and (5) above 30,000 CNY. An SES composite score was created by standardizing and averaging parental education, occupational prestige, and income, consistent with established practices in SES research (Bradley and Corwyn, 2002; Sirin, 2005). This multidimensional operationalization captures the material and human capital dimensions of family background (Conger and Donnellan, 2007). In the present sample, the modal household income category was below 5,000 CNY (*n* = 109, 48.2 percent), reflecting the participation of students from economically diverse family backgrounds.

#### 3.2.2 Cultural capital resources

Cultural capital was assessed through four indicators capturing the science-related resources available in students' home environments. Tutoring resource was measured by the frequency of attending physics tutoring outside of school on a 5-point scale. The science book collections was measured by a single item asking how many physics and science-related books the family owns (excluding textbooks) on a 5-point scale (1 = none, 2 = 1 to 5 books, 3 = 6 to 10 books, 4 = 11 to 20 books, 5 = more than 20 books). Physics daily study time investment was measured by daily study time outside of class and weekend study time, which rated on 5-point scales. Science exhibition was assessed by the frequency of parents taking the student to extracurricular science experiments or science and technology exhibitions on a 5-point scale (1 = never, 5 = very frequently). A composite cultural capital score was computed by standardizing and averaging these indicators. This operationalization aligns with Bourdieu (1986) conceptualization of objectified and institutionalized cultural capital as applied to the science learning domain (Lareau, 2011). Higher scores indicate greater access to science-related cultural resources in the home environment.

#### 3.2.3 Parental educational expectations

Parental educational expectations were assessed through items capturing parents' educational aspirations and their behavioral expressions. Homework checking was included because parents with higher educational expectations are more likely to monitor and check their children's homework to ensure that academic performance is consistent with those expectations. Parenting style was included because authoritative parenting is characterized by high expectations combined with supervision and support. Although these items are behavioral rather than direct expectation statements, they are retained as observable manifestations of parental educational expectations in the Chinese family context, where expectations are frequently communicated through daily supervision and parenting practices. This operationalization is consistent with previous research showing that parental expectations influence academic outcomes through both motivational and behavioral pathways (Davis-Kean, 2005; Jodl et al., 2001).

#### 3.2.4 Physics achievement

Physics achievement was measured by students' most recent standardized physics examination score, reported on a 5-point scale: (1) below 54 points, (2) 55 to 64 points, (3) 65 to 74 points, (4) 75 to 84 points, and (5) 85 points and above (out of 100). Standardized school examination scores have been widely used as valid indicators of domain-specific academic achievement in STEM education research (Maltese and Tai, 2011). The examination was administered uniformly across the school as part of the regular semester assessment. In the present sample, the modal score category was 85 points and above (*n* = 103, 45.6%), indicating a positively skewed distribution typical of teacher-assigned school examinations.

#### 3.2.5 Additional variables

Demographic variables, including gender, grade level, residential location, and parents' marital status, were also collected to describe the sample and serve as covariates in the analyses.

### 3.3 Procedure

Data were collected during regular class sessions under the supervision of trained research assistants. Before completing the questionnaire, participants were informed that the survey was anonymous and that all responses would be used solely for academic research purposes. Students were also told that there were no right or wrong answers and that they should respond honestly based on their actual experiences and perceptions. The questionnaire required approximately 15 to 20 min to complete. All students present during the data collection sessions voluntarily participated in the survey. No monetary compensation was provided, which is consistent with common practices in school-based educational research in China.

### 3.4 Data analysis

Data analysis was conducted in Stata 17.0. Stata was selected because it supports CFA, bootstrap mediation, interaction analysis, simple slopes, and regression diagnostics within one reproducible workflow. AMOS provides a convenient graphical interface for covariance-based SEM but is less efficient for the combined regression and bootstrap conditional-process analyses used here, while SmartPLS is primarily designed for prediction-oriented partial least-squares SEM rather than the confirmatory covariance-based approach adopted in this study.

The analysis proceeded in several stages. First, descriptive statistics and Pearson correlations were computed. Second, confirmatory factor analysis assessed the measurement model using standardized loadings, AVE, CR, MSV, and model-fit indices. Third, hierarchical OLS models estimated the SES-achievement association, the two mediators, and the interaction between SES and rural residence, with grade, gender, and parents' divorce status included as controls. The binary moderator was coded 1 for rural residence and 0 for urban residence and was mean-centered before constructing the interaction term. Fourth, parallel mediation was examined with 5,000 bootstrap resamples and bias-corrected 95% confidence intervals.

Physics achievement was recorded in five ordered score categories. Because the categories approximate an underlying continuous examination-score distribution and five-category variables can often be analyzed as approximately continuous under acceptable distributional conditions (Rhemtulla et al., 2012), the primary models used OLS with robust standard errors. To assess sensitivity to this decision, the main models were also re-estimated with ordered logistic regression; the directions and significance conclusions for SES, cultural capital, parental educational expectations, and the interaction between SES and residence were unchanged. The PROCESS macro (Model 4; Hayes, 2018) was employed with both mediators entered simultaneously, using 5,000 bootstrap resamples and bias-corrected 95% CIs.

## 4 Results

### 4.1 Descriptive statistics and preliminary analyses

Table 1 presents the means, standard deviations, and Pearson correlations for all study variables. The average family SES composite was 1.93 (SD = 1.03) on a 1–5 scale, indicating modest socioeconomic status overall. Cultural capital averaged 2.23 (SD = 1.02), suggesting limited access to science-related educational resources. Parental expectations averaged 1.91 (SD = 0.61), and physics achievement averaged 3.73 (SD = 1.04) on a 1–5 scale. Correlation analysis revealed that SES was positively associated with physics achievement (β = 0.373, *p* < 0.001), cultural capital (β = 0.161, *p* = 0.015), and parental expectations (β = 0.187, *p* = 0.005). Both cultural capital (β = 0.290, *p* < 0.001) and parental expectations (β = 0.249, *p* < 0.001) were also positively correlated with physics achievement. Urban residence showed weak correlations with all main constructs.

**Table 1:** Descriptive statistics and correlations among study variables.

Variable	*M*	SD	1	2	3	4
1. Family SES	1.93	1.03	1			
2. Cultural capital	2.23	1.02	0.161	1		
3. Parental expectations	1.91	0.61	0.187	0.067	1	
4. Physics achievement	3.73	1.04	0.373^***^	0.290^***^	0.249^***^	1

*N* = 226. ^***^ *p* < 0.001.

### 4.2 Confirmatory factor analysis

CFA results (Table 2) showed all standardized factor loadings were significant (0.834–0.947, all *p* < 0.001). Parental occupation was examined during construct development but did not load adequately with parental education and household income; it was therefore excluded from the final SES factor (Baumrind, 1966). Convergent validity was supported: AVE = 0.889 (SES), 0.748 (CC), 0.807 (PE), all exceeding the 0.50 threshold. Composite reliability was excellent: CR = 0.960 (SES), 0.935 (CC), 0.894 (PE). Discriminant validity was confirmed as all AVE values exceeded their respective MSV values (MSV: SES = 0.035, CC = 0.027, PE = 0.035), and the square root of AVE for each construct exceeded its correlations with other constructs. The measurement model demonstrated acceptable absolute fit (χ^2^(24) = 182.73, *p* < 0.001; SRMR = 0.061), though RMSEA was elevated (0.071; 90% CI [0.032, 0.095]), likely attributable to the small number of items per construct and high factor loadings (Kenny et al., 2015). Incremental fit indices were acceptable (CFI = 0.912, TLI = 0.920).

**Table 2:** Confirmatory factor analysis of studied constructs.

Variable	Loading	*t*	AVE	MSV	CR
**Family SES**			0.889	0.035	0.960
Father education	0.937	40.05			
Mother education	0.947	44.39			
Household income	0.944	43.03			
**Cultural capital**			0.748	0.027	0.935
Tutoring resources	0.893	29.81			
Daily study time	0.834	22.63			
Science exhibitions	0.901	31.23			
Science book collection	0.911	33.06			
**Parental expectations**			0.807	0.035	0.893
Homework checking (*r*)	0.898	30.64			
Parenting style	0.898	30.64			

AVE, Average variance extracted; CR, Composite reliability; MSV, Maximum shared variance; (*r*), Recoded (1 = Yes, 0 = No). Model fit index: CMIN =182.73(df = 24); SRMR = 0.061; RMSEA = 0.071; IFI = 0.91; TLI = 0.92; CFI = 0.912.

### 4.3 Regression results

Table 3 presents three OLS regression models of physics achievement, with grade, gender, and parents' divorce status as controls. Model 1 examined the total effect: SES significantly associated with physics achievement (β = 0.389, *p* < 0.001), supporting H1. Among the control variables, none reached statistical significance (Grade: β = −0.103, *p* = 0.274; Gender: β = 0.067, *p* = 0.595; Divorce: β = 0.125, *p* = 0.584). Model 2 added both mediators: cultural capital (β = 0.244, *p* < 0.001) and parental expectations (β = 0.172, *p* = 0.005) were both significant predictors, while the direct effect of SES remained significant but was reduced (β = 0.319, *p* < 0.001), suggesting partial mediation. Model 2 explained 23.8% of the variance in physics achievement (*R*^2^ = 0.238). Model 3 introduced the moderation terms: rural residence (mean-centered) and the SES × Rural interaction. The interaction term was significant (β = 0.285, *p* = 0.018), indicating that rural residence moderates the SES -> achievement relationship. In this full model, SES (β = 0.334, *p* < 0.001), cultural capital (β = 0.254, *p* < 0.001), parental expectations (β = 0.185, *p* = 0.002), and rural residence (β = 0.357, *p* = 0.004) were all significant, with the model explaining 28.4% of the variance (*R*^2^ = 0.284).

**Table 3:** The effect of SES on physics achievement.

**Independent variable**	Dependent variable (physics achievement)								
	**Model 1**			**Model 2**			**Model 3**		
	β	*t*	**VIF**	β	*t*	**VIF**	β	*t*	**VIF**
Family SES	0.389^***^	6.175	1.03	0.319^***^	5.145	1.10	0.334^***^	5.567	1.11
Cultural capital				0.244^***^	4.067	1.05	0.254^***^	4.305	1.05
Parental expectations				0.172^**^	2.820	1.07	0.185^**^	3.083	1.08
Rural							0.357^**^	2.950	1.06
Family SES × rural							0.285^*^	2.375	1.01
Gender	0.067	0.536	1.01	0.044	0.367	1.03	0.047	0.402	1.03
Grade	−0.103	1.096	1.16	−0.145	1.611	1.17	−0.104	1.169	1.21
Parents divorced	0.125	0.548	1.18	0.101	0.461	1.21	0.134	0.626	1.22
*R* ^2^	0.149			0.238			0.284		
Adj *R*^2^	0.133			0.217			0.257		
*F*	9.66^***^			11.37^***^			10.75^***^		
*N*	226			226			226		

Standardized regression coefficients are reported. ^*^ *p* < 0.05, ^**^ *p* < 0.01, ^***^ *p* < 0.001.

### 4.4 PROCESS mediation analysis

To formally test parallel mediation (H2 and H3), the PROCESS macro (Model 4; Hayes, 2018) with 5,000 bootstrap resamples and bias-corrected confidence intervals was employed, controlling for grade, gender, and parents‘ divorce status. SES significantly predicted cultural capital (path *a*1: β = 0.140, *p* = 0.038) and parental expectations (path *a*2: β = 0.210, *p* = 0.002). Both mediators significantly predicted physics achievement: cultural capital (path *b*1: β = 0.244, *p* < 0.001) and parental expectations (path *b*2: β = 0.172, *p* = 0.005). The direct effect of SES on physics achievement remained significant (*c*': β = 0.319, *p* < 0.001), indicating partial mediation. The total effect of SES on physics achievement was significant (*c*: β = 0.389, *p* < 0.001). The indirect effect through cultural capital was significant (ab = 0.034, Boot SE = 0.019, 95% BC CI [0.006, 0.084]), supporting H2. The indirect effect through parental expectations was also significant (ab = 0.036, Boot SE = 0.016, 95% BC CI [0.011, 0.076]), supporting H3. The total indirect effect was significant (ab = 0.070, Boot SE = 0.025, 95% BC CI [0.030, 0.127]).

### 4.5 Moderation by rural residence

The significant SES × Rural interaction in Model 3 (β = 0.285, *p* = 0.018) confirmed H4. The reported simple slopes analysis revealed that the positive effect of SES on physics achievement was significant in both groups but substantially stronger among rural students (β = 0.507, *t* = 5.20, *p* < 0.001) than among urban students (β = 0.222, *t* = 2.99, *p* = 0.003). The Johnson-Neyman technique identified that the SES -> achievement association was significant and positive across the full range of the urban residence variable, with the slope increasing monotonically as urban residence increased. This pattern indicates that rural residence amplifies the benefits of family SES for physics achievement, consistent with the resource amplification hypothesis.

### 4.6 Model diagnostics and robustness checks

Regression diagnostics did not indicate serious violations of the OLS assumptions. All variance inflation factors (VIF) were below the conventional threshold of 5 (range 1.01–1.22; mean VIF = 1.10), the Durbin-Watson statistic was 1.805, and the Breusch-Pagan test was non-significant (χ^2^(8) = 4.01, *p* = 0.857). The standardized achievement outcome also showed skewness (−0.192) and kurtosis (0.134) within conventional acceptable ranges.

As a robustness check, all three models were re-estimated using ordered logistic regression with the five-category physics score as the outcome. As shown in Table 4, the direction and significance of the key coefficients were unchanged: SES (β = 0.605, *p* < 0.001), cultural capital (β = 0.528, *p* < 0.001), parental expectations (β = 0.413, *p* = 0.010), rural residence (β = 0.993, *p* < 0.001), and the SES × rural interaction (β = 0.565, *p* < 0.010). Model fit was adequate (LR χ^2^(8) = 63.28, *p* < 0.001, pseudo-*R*^2^ = 0.100). Taken together, these checks indicate that the substantive conclusions were not dependent on multicollinearity, residual autocorrelation, heteroscedasticity, or treating the five-category outcome as approximately continuous.

**Table 4:** Robustness checks using ordered logistic regression.

**Independent variable**	Dependent variable (physics achievement)								
	**Model 1**			**Model 2**			**Model 3**		
	β	*t*	**VIF**	β	*t*	**VIF**	β	*t*	**VIF**
Family SES	0.624^***^	4.175	1.03	0.513^***^	3.828	1.10	0.605^***^	4.231	1.11
Cultural capital				0.494^***^	3.742	1.05	0.528^***^	3.911	1.05
Parental expectations				0.347^**^	2.776	1.07	0.413^**^	3.177	1.08
Rural							0.993^**^	3.521	1.06
Family SES × rural							0.565^**^	3.102	1.01
Gender	−0.016	0.287	1.01	−0.090	0.742	1.03	−0.109	0.431	1.03
Grade	−0.054	0.065	1.16	−0.141	0.361	1.17	−0.008	0.041	1.21
Parents divorced	0.263	0.558	1.18	0.204	0.435	1.21	0.329	0.687	1.22
LR chi^2^	24.20			47.71			63.28		
*R* ^2^	0.038			0.075			0.100		
*p* (LR chi^2^)	< 0.001			< 0.001			< 0.001		
*N*	226			226			226		

Logit coefficients are reported. ^**^ *p* < 0.01, ^***^ *p* < 0.001.

## 5 Discussion

This study examined the pathways through which family socioeconomic status influences physics achievement among Chinese high school students by testing an integrated mediation model grounded in Bourdieu's cultural capital theory and Eccles' expectancy-value theory. Using survey data collected from 226 students in Fujian Province, the results demonstrated that family SES positively predicts physics achievement, and that this relationship is partially mediated by cultural capital and parental educational expectations. In addition, urban-rural differences were observed in the relationship between family background and students' perceptions of physics learning. These findings contribute to the literature on educational inequality and STEM achievement in several important ways.

### 5.1 Theoretical contributions

First, this study extends the well-established SES-achievement relationship to the domain-specific context of physics education in China. Although previous research has consistently demonstrated that family SES positively predicts general academic achievement (Sirin, 2005; White, 1982), considerably less attention has been devoted to subject-specific STEM achievement, especially physics achievement. Physics differs from many other school subjects because it requires abstract reasoning, mathematical competence, and conceptual understanding. The present findings are consistent with recent evidence linking SES with subject-specific achievement among Chinese adolescents (Xu and Jin, 2024) and with school-level evidence connecting poverty, physical-science performance, and educational progression (Hatzfeld et al., 2025). The present findings indicate that family SES remains an important predictor even within a subject domain often perceived as highly meritocratic and ability-driven.

Second, the findings provide empirical support for Bourdieu's (1986) cultural capital theory in the context of science education. The significant mediating effect of cultural capital suggests that disparities in science-related educational resources, such as science books, study time, and participation in science activities, represent important mechanisms through which socioeconomic advantages are translated into academic advantages. This finding is consistent with previous research on science capital and science learning (Archer et al., 2012; DeWitt et al., 2016; Jin et al., 2024). More importantly, the present study demonstrates that even within a single Chinese school context, variation in science-related cultural capital significantly predicts differences in physics achievement.

Third, the mediating role of parental educational expectations supports expectancy-value theory (Eccles and Wigfield, 2002) and aligns with previous research showing that parental expectations influence students' academic motivation and achievement (Davis-Kean, 2005; Simpkins et al., 2012). In the Chinese educational context, where academic achievement is strongly associated with future social mobility, parental expectations may exert particularly strong motivational effects. This finding accords with recent Chinese research linking parental expectations with study engagement and academic success (Song et al., 2024; Wang, 2024; Ye et al., 2024; Shen et al., 2025). The present findings suggest that higher-SES parents not only possess greater material resources but also communicate stronger educational aspirations, which in turn positively influence students' engagement and achievement in physics learning.

Fourth, the urban-rural differences observed in this study contribute to the broader literature on educational inequality in China (Hannum and Park, 2007; Li et al., 2017). Rural students reported higher perceived physics difficulty and appeared more sensitive to family socioeconomic conditions. This result is consistent with recent evidence that family SES accounts for an important share of rural educational disadvantage in China (Hu, 2025) and with broader evidence that rural-urban inequalities remain sensitive to differences in family resources and access to educational opportunities (Li et al., 2024; Sun et al., 2024). This finding suggests that structural inequalities in educational resources may influence not only objective academic achievement but also students' subjective learning experiences and academic confidence.

Interestingly, students from higher-income families reported greater perceived physics difficulty despite achieving higher examination scores. This counterintuitive finding may reflect the “big-fish-little-pond effect” (Marsh, 1987), whereby students in more competitive educational environments develop higher academic standards and therefore perceive academic tasks as more challenging.

### 5.2 Practical implications

The findings have cautious implications for educational practice and policy. Because cultural capital partially mediated the SES-achievement relationship, schools may consider expanding access to science books, laboratory experiences, tutoring, school-based science clubs, and informal science activities for students with fewer family resources. These recommendations should be evaluated through longitudinal or experimental designs rather than treated as effects established by the present cross-sectional study.

Second, the importance of study time investment suggests that structured after-school physics support programs may be especially beneficial for students who lack supportive home learning environments. Schools may provide supervised study programs, physics tutoring opportunities, or peer-learning groups to support students from lower-SES backgrounds.

Third, the significant role of parental educational expectations highlights the importance of strengthening parent-school communication and parental involvement. Educational programs designed to help parents understand how to support students' science learning may enhance students' motivation and academic persistence. Importantly, such support programs should target not only highly educated parents but also families with limited educational backgrounds.

Fourth, the urban-rural differences identified in this study suggest that policy efforts should pay particular attention to educational inequality between urban and rural regions. Increasing educational investment in rural schools, improving science laboratory facilities, and strengthening teacher training in rural areas may help reduce disparities in physics learning opportunities.

Finally, educators should recognize that students' physics achievement is associated not only with individual academic skills but also with family educational resources, cultural experiences, and motivational support. A broader understanding of these correlates may inform efforts to make physics-learning opportunities more equitable.

### 5.3 Limitations and future directions

Several limitations of the present study should be acknowledged. First, the cross-sectional design does not establish temporal ordering among SES, cultural capital, parental expectations, and physics achievement. The regression and mediation results therefore represent conditional associations and statistical indirect effects rather than causal mechanisms; reverse relationships and residual confounding cannot be excluded.

Second, all participants were recruited through convenience sampling from one public senior high school in Fujian Province. The findings describe this specific school context and should not be generalized to all Chinese senior high school students. Future studies should use longitudinal, quasi-experimental, or experimental designs and recruit larger probability-based or stratified samples from multiple schools and regions.

Third, several variables, including physics achievement, cultural capital, and parental educational expectations, were measured primarily through self-report questionnaires and brief purpose-developed items. Such measures may introduce common method variance and measurement bias. Future studies should incorporate standardized achievement indicators, validated multi-item scales, and multi-informant data sources such as parent and teacher reports.

Fourth, the present study focused mainly on parallel mediation pathways and did not examine more complex psychological and contextual mechanisms underlying physics achievement. Future research may explore additional factors such as physics self-efficacy, academic self-concept, learning strategies, and teacher support, as well as more sophisticated models including serial mediation and moderated mediation frameworks.

## Data Availability

The raw data supporting the conclusions of this article will be made available by the authors, without undue reservation.
